# The Maternal Instinct

**Published:** 1909-03-27

**Authors:** 


### The Maternal Instinct.

Comparative psychology is as yet in its infancy. The necessity for collecting details with laborious accuracy by observations of animals in the natural state, and the extreme difficulty of arranging trustworthy experiments must, in a measure, account for the want of progress in this branch of science. One of the chief psychological phenomena, which interests through its close affinity to a fundamental characteristic of our own female sex, is the maternal instinct. Much has been written in praise of the affection which mothers tend to show towards their offspring: still more has been said of those in whom this attitude seems wanting. In the existing condition of civilisation moral judgment has come to regard its absence as something abnormal and unnatural. But the maternal instinct is probably one of the few fundamental characteristics of the female element which remains unaltered by lesser environmental changes and still obeys the laws of natural selection. Whatever else may still be a matter of controversy, so far as concerns the origin of sex, it is generally admitted that the female element is more inert and more receptive than the male. Since the female is, in a way, far more highly specialised for purposes of reproduction, we find that, as we rise in the scale, the interest of the mother in her offspring increases. Such fish, for example, as the salmon, which produces many thousands of eggs, provide for the continuation of their race through their extraordinary fertility. But in birds, the compensation for small production is found in the care with which the young are fed until the time when they can fend for themselves. In mammals the maternal instinct is proportional to the number of offspring. Whereas we find among rabbits and mice the frequent occurrence of cannibalism on the part of the mother, such cases become more rare in the carnivora, and among men infanticide is comparatively rare.

Like her essential physical element, woman is receptive, and we make no doubt, that her primary function in life, as the result of long evolution, is to bear children, to bring them up and to launch them into the world as useful citizens. When civilisation produces great variation in work or amusement it becomes a case of priority of stimulus. The maternal instinct is the basic instinct of woman, but it need not necessarily be directed towards the nurture of her young. The stimulus is given, be it for literature, politics, or intrigue, and the woman is driven by the very force of her nature to espouse the cause which first, so to speak, fertilises her brain. The difference between man and the dog, in this respect, is that of variety of interest and consciousness of self; but the impelling instinct is there all the same. It is difficult often to reconcile natural and artificial selection, but as both phenomena form a part of nature as a whole there can be no doubt that allowance has been made in nature for the apparent freedom of choice which is granted to man. The preservation of the race does not depend upon the blind instinct which causes the bird to make a nest or the lioness to fight for her young, but upon the more or less intelligent selection by woman of the means at her disposal for the education of her young. Hence we should expect to find this instinct directed into other channels, and in highly civilised countries a decrease in population. The increased percentage of female births in cities is a natural compensation for the corresponding increase of variety in the matter of interests. The more highly domesticated animals exemplify in a lesser degree this gradual deflection of the primary instinct. While mice very frequently take no notice whatever of their young and leave them to die of inanition, cats and dogs which are entirely with men, often make but little display when their young are removed. Yet if from causes more or less artificial there are lesser births among civilised races there should be a compensating reduction, by artificial means if necessary, in infant mortality; and this is precisely what is taking place in the more enlightened and far-seeing among the nations. Great efforts are being made to render the infant's struggle for existence as slight as possible at the outset—a course which is obviously compensatory for the change in the woman's attitude, and perhaps the only effectual counterblast to the artificial limitation of families.

